# Preserving beta cell function in children and adolescents with newly diagnosed stage 3 type 1 diabetes: per-protocol population analysis from the PROTECT randomised trial

**DOI:** 10.1007/s00125-026-06849-5

**Published:** 2026-09-10

**Authors:** Kevan C. Herold, Colin M. Dayan, Lucienne Chatenoud, Stephen E. Gitelman, Zdenek Sumnik, Kimber Simmons, Agnieszka Szypowska, Laura A. Knecht, Elisabeth Niemoeller, Mark R. Rigby, Wei Tian, Robert Hill, Eleanor L. Ramos

**Affiliations:** 1 Department of Immunobiology, Yale School of Medicine, New Haven, Connecticut USA; 2 Department of Endocrinology, Cardiff University School of Medicine, Cardiff, UK; 3 Université Paris Cité, CNRS, Inserm, Institut Necker Enfants Malades-INEM, Paris, France; 4 Department of Pediatrics, University of California San Francisco, San Francisco, CA USA; 5 Department of Pediatrics, Motol University Hospital and 2nd Faculty of Medicine, Prague, Czechia; 6 Barbara Davis Center for Diabetes/University of Colorado School of Medicine, Aurora, CO USA; 7 Department of Pediatric Diabetology and Paediatrics, Medical University of Warsaw, Warsaw, Poland; 8 Sanofi, Morristown, NJ USA; 9 Sanofi, Industrial Park Höchst, G879, Frankfurt am Main, Germany; 10 Provention Bio, Morristown, NJ USA

**Keywords:** Clinical immunology, Diabetes in childhood, Prediction and prevention of type 1 diabetes

## Abstract

**Aims/hypothesis:**

In the PROTECT randomised trial, teplizumab demonstrated significantly greater beta cell function preservation vs placebo in the intent-to-treat (ITT) population. Analyses of the pre-specified per-protocol (PP) population and PP subgroups are presented to further evaluate the efficacy of teplizumab.

**Methods:**

Participants aged 8–17 years with stage 3 type 1 diabetes with a peak stimulated C-peptide level ≥0.2 nmol/l (ITT population: *N*=328) were randomised 2:1 to receive two 12-day courses of teplizumab or placebo intravenously. Participants with <80% treatment adherence, who took prohibited medication, received incorrect study treatment or were pregnant were excluded from the PP population. Subgroup analyses were based on the presence of HLA-DR3 or HLA-DR4 and the type of baseline type 1 diabetes autoantibodies.

**Results:**

The PP population comprised 275 participants (teplizumab, *n*=180; placebo, *n*=95). At week 78, the primary endpoint of the least squares (LS) mean difference in change of stimulated C-peptide concentration from baseline between teplizumab and placebo was 0.14 nmol/l (95% CI 0.10, 0.18; *p*<0.001). At week 78, teplizumab treatment significantly reduced the exogenous insulin dose (LS mean difference –0.17 U kg^−1^ day^−1^; 95% CI –0.26, –0.08; *p*<0.001) and increased the time in range (TIR) (LS mean difference 6.17%; 95% CI 0.13, 12.2; *p*<0.05) vs placebo. In subgroup analyses, treatment with teplizumab resulted in higher C-peptide levels compared with placebo, regardless of HLA-DR3/HLA-DR4 presence or baseline type 1 diabetes autoantibodies.

**Conclusions/interpretation:**

Teplizumab treatment per protocol led to greater beta cell function preservation, greater TIR and lower exogenous insulin use compared with placebo. Beta cell function preservation was maintained irrespective of HLA subtype or the presence of type 1 diabetes autoantibodies.

**Trial registration:**

ClinicalTrials.gov NCT03875729

**Funding:**

The study was funded by Provention Bio, a Sanofi company

**Graphical Abstract:**

**Figure:**
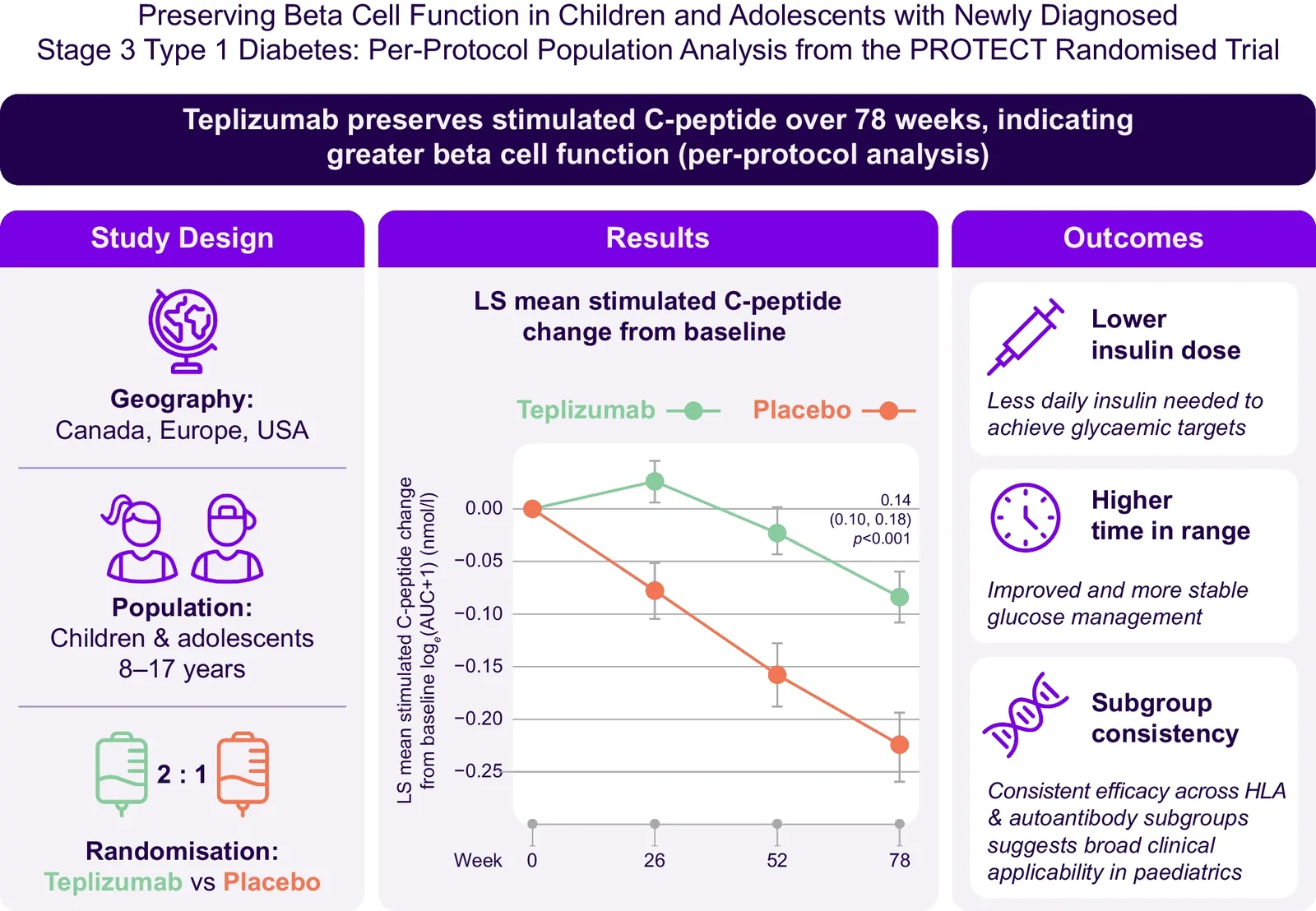

**Supplementary Information:**

The online version of this article (https://doi.org/10.1007/s00125-026-06849-5) contains peer-reviewed but unedited supplementary material.

**Figure:**
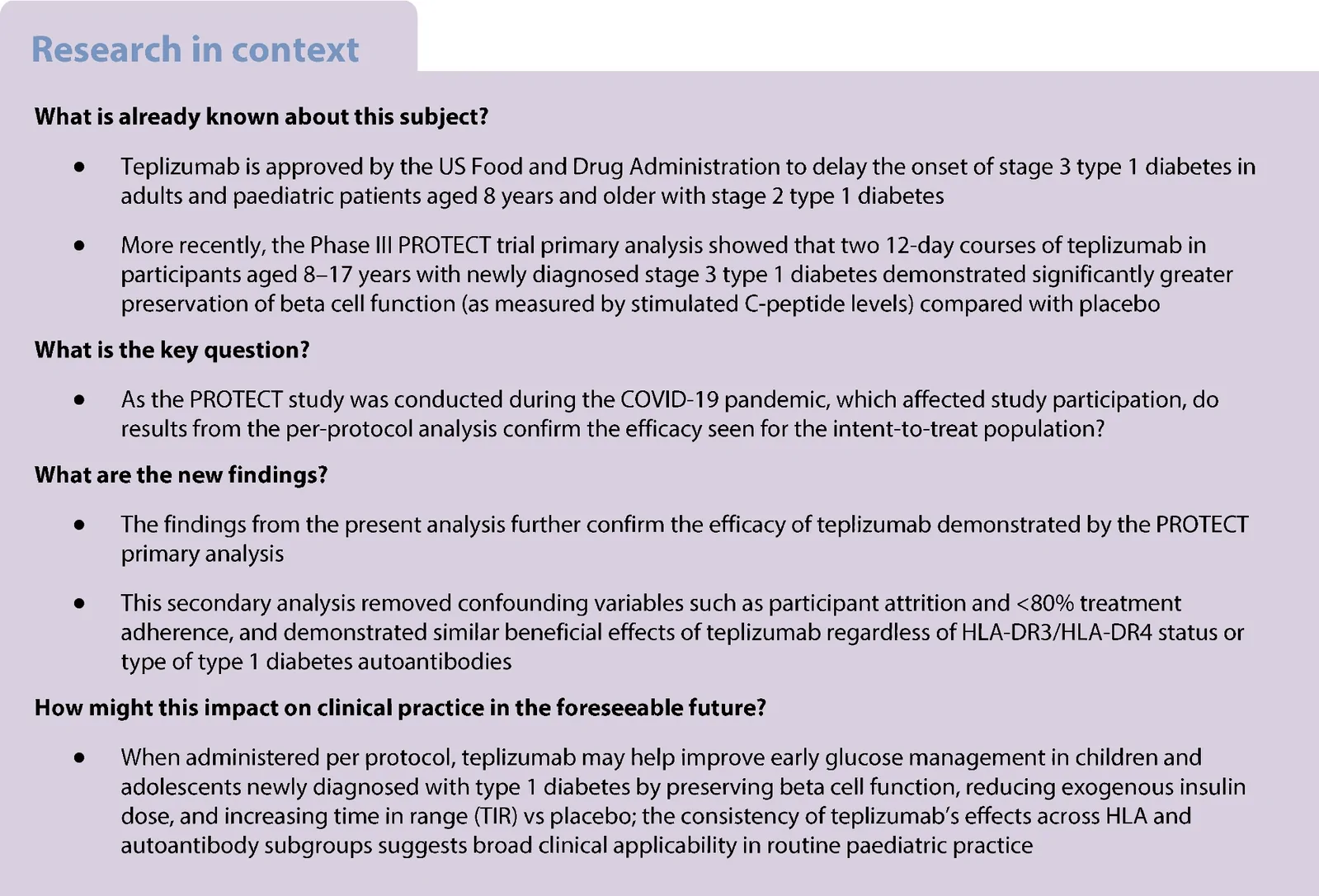

## Introduction

In type 1 diabetes, the autoimmune-mediated destruction of insulin-producing beta cells leads to the requirement for exogenous insulin to regulate metabolic control and ensure survival of the individual [1, 2]. Despite recent technological advances, the burden of disease is high in children and young people, who often experience accelerated disease progression and often have difficulty reaching the target HbA_1c_ levels recommended by the American Diabetes Association and other medical societies [3–7]. Furthermore, individuals with type 1 diabetes can experience hypoglycaemic events that may be severe or life-threatening and can contribute to defective glucose counter-regulation in the long-term. As such, glycaemic management in children and young people remains difficult to achieve, and constitutes a substantial unmet need in the treatment of type 1 diabetes [8]. It has been suggested that retention of residual endogenous insulin production may improve glycaemic management and reduce the risk of hypoglycaemia induced by the use of exogenous insulin [9, 10].

Teplizumab, an Fc receptor-non-binding anti-CD3 monoclonal antibody, received approval from the US Food and Drug Administration in 2022 as a treatment to delay onset of stage 3 type 1 diabetes in people aged ≥8 years old with stage 2 type 1 diabetes [11]. In the primary analysis of the intent-to-treat (ITT) population in the PROTECT trial (NCT03875729) of children and adolescents with newly diagnosed type 1 diabetes, treatment with two 12-day courses of teplizumab (*n*=217) achieved significantly higher stimulated C-peptide levels compared with placebo [12]. These data indicate that teplizumab treatment has significant benefit in the maintenance of beta cell insulin production [12]. Furthermore, at week 78, a clinically meaningful peak C-peptide level (≥0.2 nmol/l) was maintained in 94.9% of participants treated with teplizumab, compared with 79.2% of those receiving placebo [12].

The PROTECT study was performed during the COVID-19 pandemic, which presented unpredictable restrictions to trial participation, and prevented some participants from completing the study per protocol (PP). Those individuals were included in the ITT population analysis, but their outcomes may have been affected by reduced study participation. Here we present findings for the PP population analysis from the PROTECT study, which are especially important in light of the effects of the COVID-19 pandemic. The pre-specified PP population analysis further explored the efficacy of teplizumab, removing the confounding variables of participant attrition and treatment administration issues [13, 14]. Additionally, we present post hoc subgroup analyses of the PP population for those participants with and without human leukocyte antigen (HLA) DR3 and DR4, and by type of type 1 diabetes autoantibodies at baseline, to examine their effect on teplizumab efficacy.

## Methods

### Study design and participants

Full methods for the PROTECT trial have been reported previously [12]. In brief, PROTECT was a multicentre, double-blind, randomised, trial assessing the ability of teplizumab to preserve beta cell function and improve clinical variables compared with placebo in newly diagnosed children and adolescents aged 8–17 years with stage 3 type 1 diabetes diagnosed within 6 weeks prior to enrolment. The study was conducted at 61 sites in the USA, Canada and Europe from March 2019 until May 2023. Eligibility criteria included the presence of at least one type 1 diabetes-associated autoantibody (GAD65 antibody, IA-2A, ZnT8A, islet cell cytoplasmic antibody or IAA) and a peak stimulated C-peptide of ≥0.2 nmol/l. Sex/gender were determined by self-report, and were included in the statistical analyses. There are no firm data on gender difference in type 1 diabetes. Male individuals appear to be more frequently affected by the disease, although this may vary between populations. There were no exclusions based on gender, sex, ethnicity or race. The patient population was considered to be similar to those in other clinical trials of type 1 diabetes, although most participants were White, which is not representative of the general population, and had lower rates of diabetic ketoacidosis than other studies in new-onset disease [12].

The pre-specified PP population excluded participants as described in electronic supplementary material (ESM) Table 1. The exclusion was done before unblinding. Treatment adherence within each course was calculated as the total amount of drug administered (μg) divided by the total amount of drug prescribed (μg) × 100, during the dosing period. Treatment adherence was tabulated in categories of <80% and ≥80%. Participants and/or their appropriate legal guardian provided informed consent and/or assent, and the study was conducted according to the International Council for Harmonisation good clinical practice guidelines and the ethical principles of the Declaration of Helsinki. The trial is registered with ClinicalTrials.gov (NCT03875729).

### Randomisation and masking

Randomisation into treatment and placebo arms for the PROTECT trial has been reported in detail previously [12]. Briefly, eligible participants were randomised (2:1) to receive two 12-day courses of teplizumab or matching placebo intravenously.

### Procedures

The trial procedures have been published previously [12]. Participants assigned to teplizumab were to receive two 12-day courses of intravenous teplizumab daily, 26 weeks (6 months) apart (day 1: 106 μg/m^2^; day 2: 425 μg/m^2^: day 3–12: 850 μg/m^2^) or placebo in an inpatient/outpatient setting based on study site procedures. Doses of the study drug were calculated based on the participant’s body surface area (square root [height in cm × weight in kg divided by 3600]), measured on the first day of each treatment course. Due to COVID-19 pandemic-related restrictions, the protocol was amended to include a modified dosing schedule to allow the second 12-day course to be administered 12 months after randomisation for participants who were unable to receive the second course at 6 months as originally planned. This delay in dosing was not a reason for exclusion from the PP analysis. Participants were administered a non-steroidal anti-inflammatory drug or paracetamol (acetaminophen) and an antihistamine and/or an anti-emetic, at least 30 min prior to administration of the study treatment (teplizumab or placebo), for at least the first 5 days of each study treatment course.

Participants were provided with a continuous glucose monitoring (CGM) device (Dexcom G6) that captured data at seven defined timepoints, for 14 days each time. The 14‑day period reflects the CGM data‑collection window rather than the wear time of a single Dexcom G6 sensor. The CGM data were used to calculate time in range (TIR; glucose 3.9–10 mmol/l [70–180 mg/dl]) and other standard CGM metrics, including time above range (>10 mmol/l [>180 mg/dl]) and time below range (<3.9 mmol/l [<70 mg/dl]). Insulin dose and clinically important hypoglycaemic events were documented in an electronic diary (eDiary), and were also recorded as adverse events (AEs) by the investigators in the AE electronic case report form. Hypoglycaemic events were collected by study teams throughout the course of the trial, and participants and their families were encouraged to report them via the eDiary. Clinical data were reviewed by an independent data monitoring committee. Safety and tolerability outcomes from the PROTECT trial have been reported previously [12].

### Efficacy endpoints

The primary efficacy endpoint of the PP population analysis was a comparison of the change from baseline in stimulated C-peptide concentration at week 78, as measured by the area under the concentration–time curve (AUC) using the trapezoidal rule during a 4 h mixed meal tolerance test. The C-peptide AUC was analysed and transformed to log_*e*_ (natural logarithm)(AUC+1) values unless otherwise specified. Key secondary endpoints at week 78 included change from baseline for mean HbA_1c_ levels, mean daily insulin dose (U kg^−1^ day^−1^), mean daily percentage TIR, and clinically important hypoglycaemic events (defined as the total number of episodes with a blood glucose level <3.0 mmol/l [<54 mg/dl] and/or episodes of severe cognitive impairment requiring external assistance for recovery [15], such as seizure, syncope or severe confusion with or without a confirmatory low blood glucose reading), as recorded in the eDiary.

### Statistical analysis

In the PP population, the primary endpoint and secondary endpoints described in the preceding section were pre-specified, and were the same as for the ITT population [12]. Treatment differences between the teplizumab and placebo groups were assessed using ANCOVA for C-peptide log_*e*_(AUC+1), insulin dose, percentage of TIR and HbA_1c_ levels at week 78 using the covariates age group and baseline C-peptide log_*e*_(AUC+1). Under the assumption that data were not missing at random, a pattern-mixture model was used to impute missing data for these primary and secondary endpoints. The rate of clinical important hypoglycaemic events was compared between groups using a negative binomial model.

Post hoc analyses of the PP population were also conducted for the primary endpoint with stratification by dosing status (original or modified dosing schedule) as well as analysis using a mixed-effect model for repeated measures (MMRM). Clinically important hypoglycaemic events were obtained from the eDiary and AE reporting, with duplicate records removed.

Post hoc subgroup analyses included change from baseline for stimulated C-peptide concentration at week 78 by HLA-DR3 and HLA-DR4 status (positive/negative) and by ZnT8A, GAD65 antibody, IA-2A, ICA antibody and IAA status (positive/negative) for the PP population.

Statistical analyses were performed in an environment that was compliant with the US Code of Federal Regulation Part 11, using SAS Viya software, version 9.4 [16].

## Results

### Participants

The ITT population for the PROTECT trial included 328 children and adolescents who were randomised to receive teplizumab (*N*=217) or placebo (*N*=111) [12]. Of these, 275 participants were included in the PP population analysis (teplizumab, *n*=180; placebo, *n*=95, ESM Fig. 1). The primary reason for exclusion from the PP analysis (ESM Table 1) was treatment adherence <80%, representing 15% (*n*=32) and 14% (*n*=15) losses of teplizumab and placebo participants in the ITT population, respectively. Approximately one-half of the 32 participants who received <80% of the total teplizumab dose did so due to protocol-defined stopping criteria. More than one-third of the teplizumab-treated participants with <80% adherence, and a higher proportion of placebo-treated participants (approximately three-quarters), withdrew consent. The excluded participants were from 26 of the 61 study sites.

Participant demographics in the PP population were generally well balanced across the treatment arms (Table 1) and were similar to those of the ITT population [12]. The mean age of all PP participants was 12.2 years, and more than half (59%) were male. The mean time from diagnosis of type 1 diabetes was 5.3 weeks. The C-peptide AUC at baseline was similar in the two groups. Four participants in the placebo group had a history of diabetic ketoacidosis.

**Table 1:** Baseline and demographic characteristics (PP population)

	Teplizumab (*N*=180)	Placebo (*N*=95)
Age, years	12.1 ± 2.5	12.4 ± 2.5
Age group		
8–11 years	70 (39)	36 (38)
12–17 years	110 (61)	59 (62)
Male sex	103 (57)	60 (63)
Race		
White	160 (89)	79 (83)
Black or African American	4 (2)	6 (6)
Asian	3 (2)	3 (3)
Other^a^	13 (7)	7 (7)
Ethnicity		
Hispanic or Latino	12 (7)	3 (3)
Not Hispanic or Latino	161 (89)	87 (92)
Not reported	7 (4)	5 (5)
Weight, kg	47.0 ± 14.7	49.5 ± 15.5
BMI, kg/m^2^	18.8 ± 3.3	19.0 ± 3.4
Time from diagnosis^b^, weeks	5.3 ± 0.8	5.2 ± 0.8
Peak C-peptide level, nmol/l		
0.2–0.7	77 (43)	41 (43)
>0.7	103 (57)	54 (57)
C-peptide AUC^c^, nmol/l	0.74 ± 0.33	0.72 ± 0.32
HbA_1c_		
mmol/mol^d^	74 ± 18.1	78 ± 21.5
%	8.96 ± 1.66	9.27 ± 1.97
Insulin dose at baseline^e^, U kg^−1^ day^−1^	0.45 ± 0.31	0.38 ± 0.25

Values for categorical variables are *n* (%); those for continuous variables are mean  ±  SD. Percentages are based on the number of patients in each treatment group with non-missing observations. Baseline is defined as the most recent value collected prior to the first dose of the study drug

^a^The ‘Other’ race category includes one Native Hawaiian or Other Pacific Islander in the placebo group and one American Indian or Alaskan Native in the teplizumab group; four participants in the teplizumab group who reported more than one race (and were counted only once); one and three participants in the placebo and teplizumab groups, respectively, who reported their race as ‘Other’; and five participants in each group who did not report their race

^b^Time from type 1 diabetes diagnosis to randomisation

^c^The area under the time-concentration curve (AUC) of C-peptide was computed using the trapezoidal rule after a 4 h mixed meal tolerance test

^d^HbA_1c_ results were captured as percentages; percentages were converted to mmol/mol using the standard International Federation of Clinical Chemistry formula: (HbA_1c_ % − 2.15) × 10.929=mmol/mol (available at https://ngsp.org/convert1.asp). SD was converted using a similar resource (https://ngsp.org/convert2.asp)

^e^Insulin use at baseline was calculated as the average daily use for participants who had at least 3 days of insulin use recorded in the eDiary prior to the start of study drug (*n*=101 for the teplizumab group; *n*=56 for the placebo group)

### Efficacy

#### Effects on beta cell function

**Fig. 1:**
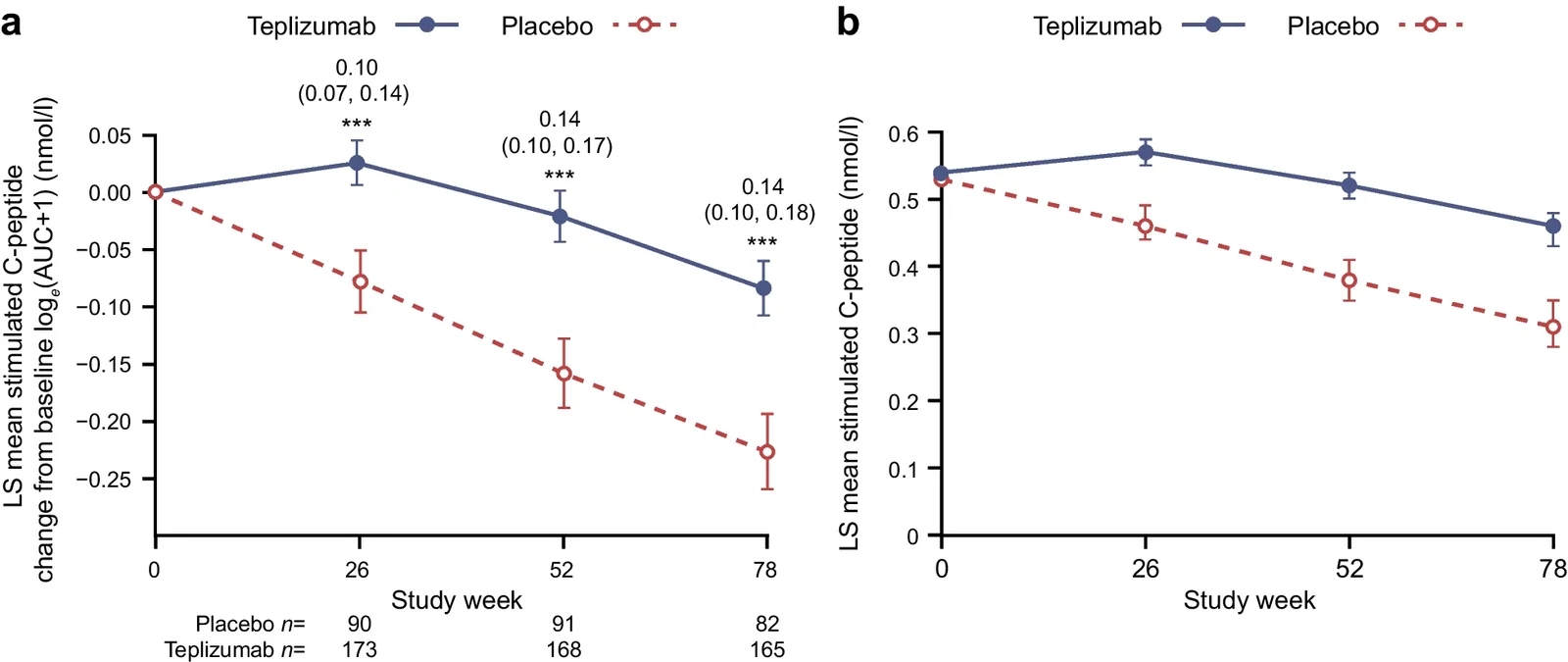
(**a**) LS mean stimulated C-peptide change from baseline log_*e*_(AUC+1) (nmol/l) and (**b**) LS mean stimulated C-peptide (nmol/l) by study week (PP population). Baseline was defined as the most recent value collected prior to the first dose of teplizumab or placebo. Estimates are based on an MMRM model with treatment group, visit, age group at randomisation and baseline C-peptide log_*e*_(AUC+1) as fixed effects, and a treatment-by-visit interaction term. The model was fitted using an unstructured covariance structure. The LS mean differences (with 95% CI) between placebo and teplizumab, and indication of significant nominal *p* values, are shown at each timepoint. ***p<0.001

In the PP population, the mean change from baseline for stimulated C-peptide AUC was significantly less at week 78 for participants treated with teplizumab (−0.08 nmol/l) compared with those treated with placebo (−0.22 nmol/l) (least squares [LS] mean difference 0.14 nmol/l, 95% CI 0.10, 0.18; nominal *p*<0.001; Table 2), representing a 64% difference (0.14/0.22 × 100) between the groups. In the teplizumab group, the stimulated C-peptide levels were greater than in the placebo group for all post-baseline timepoints (Fig. 1). An MMRM sensitivity analysis of the primary endpoint revealed a significantly greater change from baseline for C-peptide levels at week 78 for participants receiving placebo compared with those receiving teplizumab (*p*<0.001), in agreement with the results of the pre-specified PP analysis.

**Table 2:** Efficacy endpoints (PP population)

	Teplizumab (*N*=180)		Placebo (*N*=95)		LS mean difference between teplizumab and placebo	Estimated rate ratio	Nominal *p* value
	Mean ± SD	*n*	Mean ± SD	*n*			
Primary endpoint							
C-peptide log_*e*_(AUC+1)^a,b^, nmol/l							
Baseline	0.54 ± 0.18	180	0.53 ± 0.18 ±	95	–	–	
Week 78	0.46 ± 0.19	165	0.33 ± 0.21	82	–	–	
LS mean change from baseline, nmol/l	–0.08 (–0.10, −0.06)		–0.22 (–0.25, −0.19)		0.14 (0.10, 0.18)	–	<0.001
Secondary endpoints							
HbA_1c_, mmol/mol							
Baseline	74.4 ± 18.1	180	77.8 ± 21.5	94	–	–	
Week 78	52.4 ± 13.4	168	54.3 ± 16.2	92	–	–	
HbA_1c_^a^^,c^, %							
Baseline	8.96 ± 1.66	180	9.27 ± 1.97	94	–	–	
Week 78	6.95 ± 1.23	168	7.12 ± 1.48	92	–	–	
LS mean change from baseline, %	–2.07 (–2.27, −1.87)		–1.94 (–2.21, −1.67)		–0.13 (–0.46, 0.20)	–	0.454
Exogenous insulin dose^a,d^, U kg^−1^ day^−1^							
Baseline	0.45 ± 0.31	101	0.38 ± 0.25	56	–	–	
Week 78	0.44 ± 0.25	86	0.60 ± 0.34	45	–	–	
LS mean at week 78, U kg^−1^ day^−1^	0.45 (0.39, 0.50)		0.61 (0.54, 0.69)		–0.17 (–0.26, –0.08)	–	<0.001
Clinically important hypoglycaemic events^e,f^	5.03 ± 9.23	180	4.83 ± 9.95	95	–		
Estimated rate	5.04 (3.94, 6.44)		4.63 (3.31, 6.49)		–	1.09 (0.72, 1.65)	0.690
Post hoc endpoints^e,g^							
Clinically important hypoglycaemic events (level 2 or level 3)	10.39 ± 16.59	180	13.24 ± 23.44	95			
Estimated rate	10.59 (8.54, 13.12)		11.86 (8.83, 15.94)		–	0.89 (0.62, 1.29)	0.543
Level 3 hypoglycaemic events	0.20 ± 0.77	180	0.75 ± 3.06	95			
Estimated rate	0.17 (0.10, 0.31)		0.76 (0.39, 1.46)		–	0.23 (0.09, 0.56)	0.001

Values in parentheses are 95% CI. Baseline was defined as the most recent value collected prior to the first dose of teplizumab or placebo

^a^Missing data at week 78 were multiply imputed using a pattern-mixture model under the ‘missing not at random’ assumption

^b^Estimates were obtained from an ANCOVA model that included treatment, age group at randomisation and baseline C-peptide log_*e*_(AUC+1) as independent variables

^c^Estimates were obtained from an ANCOVA model that included treatment, age group at randomisation and screening peak C-peptide category as independent variables

^d^Estimates were obtained from an ANCOVA model that included treatment, age group at randomisation, screening peak C-peptide category and baseline HbA_1c_ (%) as independent variables

^e^A clinically important episode is defined as a blood glucose <3.0 mmol/l (<54 mg/dl) (i.e. level 2 hypoglycaemia [15]) or a hypoglycaemia event of severe cognitive impairment requiring external assistance (i.e. level 3 hypoglycaemia [15]). The event rate is calculated for each participant as number of events/total trial follow-up time. The total trial follow-up time is calculated as (date of last trial contact – the first dose date + 1)/365.25. Values represent the number of events per patient-year

^f^Includes hypoglycaemic events from the eDiary only

^g^The post hoc sensitivity analysis includes hypoglycaemic events from the eDiary and AE data, with duplicate records removed. This analysis classified the reported AEs according to the Common Terminology Criteria for Adverse Events (CTCAE) grading

#### Metabolic control in the study arms

**Fig. 2:**
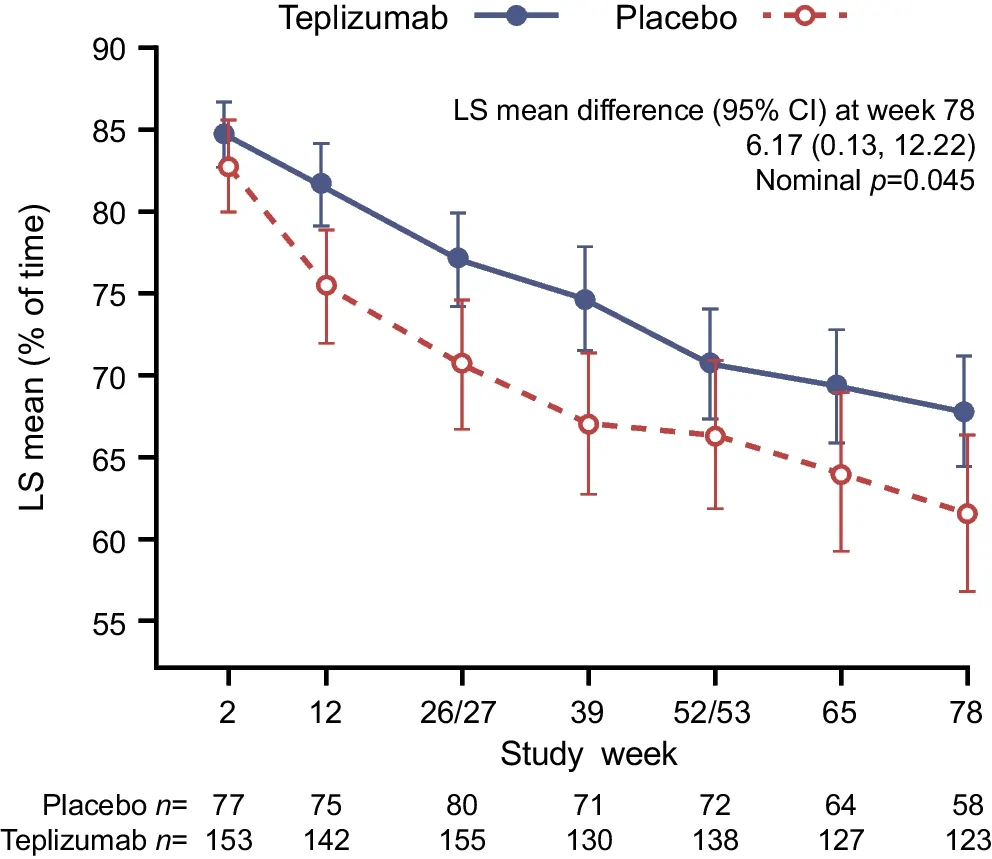
LS mean adjusted TIR (%) for glucose level management assessed using CGM by study week (PP population). TIR for glucose level management is defined as the daily average percentage of time that a participant’s glucose is 3.9–10 mmol/l [70 mg/dl–180 mg/dl], and is calculated based on participants who have at least 3 days of CGM data recorded for each visit with a range of at least 8 h on a given day. The LS mean difference estimates were obtained from an ANCOVA model that included treatment, age group at randomisation and screening peak C-peptide category as independent variables. Missing data were multiply imputed using a pattern-mixture model under the ‘missing not at random’ assumption. LS means and 95% CIs are based on an MMRM model with treatment group, visit, age group at randomisation, screening peak C-peptide category, and a treatment-by-visit interaction term as fixed effects. The model was fitted using an unstructured covariance structure. Participants under the normal dosing schedule reported CGM results for weeks 2, 12, 27, 39, 52, 65 and 78. Participants who received the modified dosing schedule reported CGM results for weeks 2, 12, 26, 39, 53, 65 and 78

As per the protocol directions to the investigators, the participants were treated to the same glycaemic target, and thus the difference in mean HbA_1c_ at week 78 was not significant between the treatment groups (52 mmol/mol [7.0%] for teplizumab vs 54 mmol/mol [7.1%] for placebo; nominal *p*=0.454). However, to achieve the same level of glucose management, the teplizumab-treated participants received less exogenous insulin compared with placebo (LS mean difference −0.17, 95% CI −0.26, −0.08; nominal *p*<0.001) (Table 2). CGM data showed a greater glucose TIR at week 78 for teplizumab compared with placebo (difference of 6.17%; 95% CI 0.13, 12.2; nominal *p*<0.05; Fig. 2).

Rates of clinically important hypoglycaemic events reported in the eDiary (secondary endpoint) were not significantly different between the teplizumab and placebo groups (Table 2). No level 3 hypoglycaemic events were reported in the eDiary, but the number of reported level 3 hypoglycaemic events based on sensitivity analysis of AEs according to the Common Terminology Criteria for Adverse Events (CTCAE) version 5.0 [17] grading showed a significant difference (nominal *p*=0.001) in favour of the teplizumab-treated group. A sensitivity analysis showed no significant difference between groups for level 2 or 3 hypoglycaemic events.

**Fig. 3:**
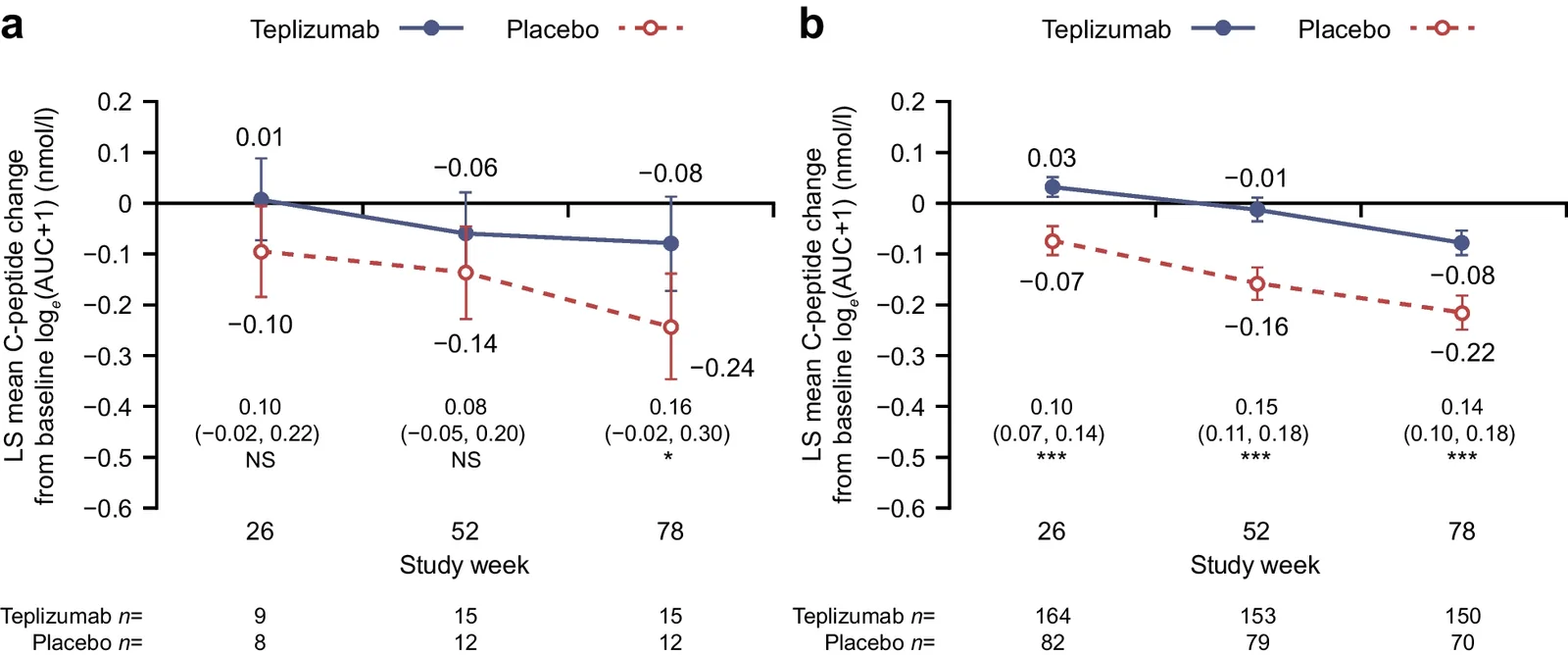
LS mean change from baseline for C-peptide log_*e*_(AUC+1) (nmol/l) for the modified dosing schedule (**a**) and planned dosing schedule (**b**) (PP population). LS mean differences (95% CI) between placebo and teplizumab, and indication of significant nominal *p* values, are shown at each timepoint. Baseline was defined as the most recent value collected prior to the first dose of the study drug. Missing data are multiply imputed using a pattern-mixture model under the ‘missing not at random’ assumption. Estimates and the *p* value are obtained from an ANCOVA model that included treatment, age group at randomisation and baseline C-peptide log_*e*_(AUC+1) as independent variables. *p<0.05; ***p<0.001; NS, not significant

Post hoc analyses of the PP population showed that the change from baseline for C-peptide levels at week 78 for participants who received a modified dosing schedule (with the second course of study drug/placebo administered at month 12 rather than month 6; teplizumab *n*=15, placebo *n*=12) was similar to those who did not (teplizumab *n*=165, placebo *n*=83) with treatment differences in favour of teplizumab of 0.16 and 0.14 nmol/l, respectively (Fig. 3). The lymphocyte count during each dosing period is shown in ESM Fig. 2.

**Fig. 4:**
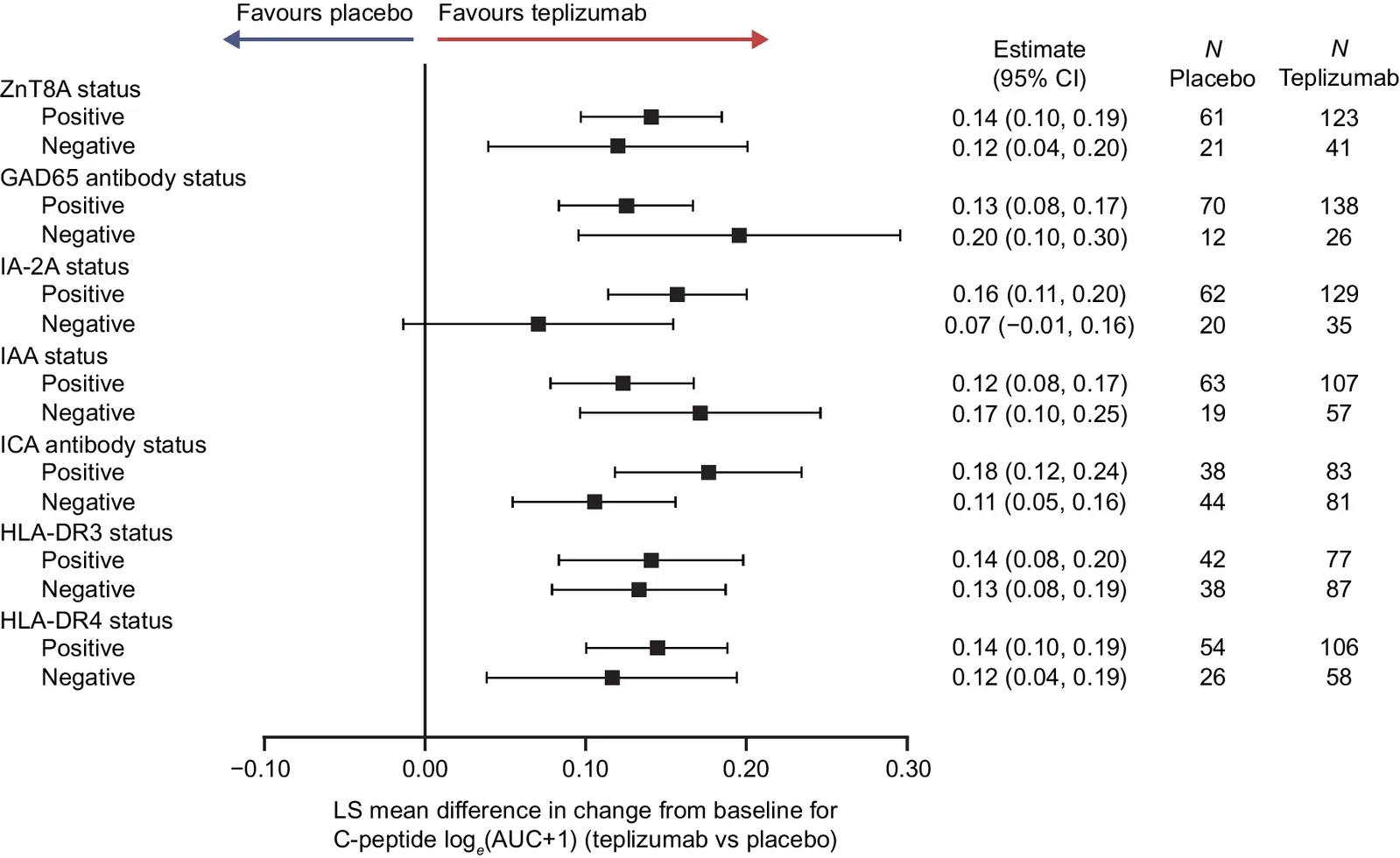
Subgroup analysis of change from baseline for C-peptide log_*e*_(AUC+1) at week 78 (PP population) according to type 1 diabetes-associated autoantibody status and HLA subtype. Baseline was defined as the most recent value collected prior to the first dose of teplizumab or placebo. Missing data at week 78 were multiply imputed using a pattern-mixture model under the ‘missing not at random’ assumption. Estimates were obtained from an ANCOVA model that included treatment, age group at randomisation and baseline C-peptide log_*e*_(AUC+1) as independent variables

In post hoc subgroup analyses of the PP population, a consistent treatment effect of teplizumab on C-peptide levels at week 78 was seen irrespective of HLA-DR3 and HLA-DR4 status or ZnT8A, GAD65 antibody, IA-2A, ICA antibody and IAA status (Fig. 4).

## Discussion

The results presented here for PROTECT participants who received at least 80% of the PP drug dose further support the clinical efficacy of teplizumab treatment. Of those excluded from the PP analysis because they had treatment adherence <80%, approximately one-half met protocol-defined stopping criteria. In addition to the differences in C-peptide, teplizumab treatment was also associated with greater TIR compared with placebo (as calculated from CGM data) and lower insulin dose. Participants receiving teplizumab had fewer level 3 hypoglycaemic events per patient-year compared with those receiving placebo, which is consistent with the higher proportion of teplizumab-treated participants maintaining higher C-peptide levels by week 78. In this PP population analysis, the mean difference in change from baseline for stimulated C-peptide AUC at week 78 between teplizumab and placebo was 0.14 nmol/l (*p*<0.001), which is similar to the treatment effect detected (LS mean difference 0.13 nmol/l; *p*<0.001) for the ITT population [12]. The results of the MMRM sensitivity analysis were in agreement with those observed in the primary analysis, demonstrating the robustness of the data. These results have particular importance because preservation of beta cell function that may translate to insulin dose maintenance for at least 78 weeks represents a clinically meaningful benefit for people with stage 3 type 1 diabetes.

In addition to the similar mean change from baseline for C-peptide AUC at week 78 between the PP and ITT populations, we did not find, in either analysis, any difference in the outcome at week 78 for the modified dosing schedule (i.e. between those who received the second course of drug at week 26 and those who received it at week 52).

It is interesting to note that, compared with the increase in C-peptide level at 6 months, the C-peptide trajectory does not change after the second course of teplizumab, whether administered at 6 or 12 months. It is likely that the initial course of teplizumab induces a functional recovery in residual beta cells by suppressing active autoimmunity. By 6 months, the beta cell reserve is reduced, and the disease is less dynamically inflammatory, thus we speculate that re-treatment primarily stabilises the trajectory rather than producing a second measurable increase in C-peptide. However, we cannot conclusively determine the effects of the second course of drug treatment based on these data. A formal comparison of one vs two courses of treatment was not performed in this study.

In this PP analysis, additional clinical benefits to those observed in the ITT analysis were identified. At week 78, HbA_1c_ levels were similar across analyses (52 mmol/mol [7.0%] for teplizumab vs 54 mmol/mol [7.1%] for placebo), and were not significantly different between treatment groups, indicating that the investigators could effectively ‘treat-to-target’, as prescribed in the protocol. However, we found more pronounced treatment differences in the PP analysis than observed in the ITT population in terms of lower insulin use in the teplizumab-treated participants, together with higher percentages for TIR at week 78 [12]. Taken together, these results indicate improved glucose management with teplizumab vs placebo.

Differences in teplizumab treatment response based on HLA and autoantibody status have been previously reported in a study of 76 relatives at risk for type 1 diabetes, although the validity of the findings may be limited by the small cohort size [18]. The study found a more robust response to teplizumab among participants who were HLA-DR4-positive vs HLA-DR4-negative, those who were HLA-DR3-negative vs HLA-DR3-positive, and those who were ZnT8A-negative vs ZnT8A-positive; however, no difference in response was observed based on the presence or absence of other autoantibodies [18]. In contrast, a prior study with teplizumab in 52 newly diagnosed participants with type 1 diabetes showed that the presence of autoantibodies (including GAD65 antibody and ZnT8A) was not predictive of treatment response [19]. Our analyses of participant subgroups in the PP population demonstrated that teplizumab efficacy was maintained in all participants irrespective of HLA-DR3 and HLA-DR4 status, as well as the type of type 1 diabetes autoantibodies.

The AEs associated with administration of teplizumab that were previously reported in the ITT analysis were consistent with those reported across the clinical development programme with teplizumab, and typically resolved at the end of the dosing regimen [12]. For example, the pattern and magnitude of lymphocyte decline in the PP population were similar to that in the ITT analysis, occurring at the same time and with a similar time to recovery. However, AEs did lead to the inability to complete the full dosing regimen in 15 participants from the ITT population (41% of those excluded in the PP analysis). As these events led to drug discontinuation but were all transient, restarting drug dosing may be considered [12]. The AEs leading to exclusion from the PP population were consistent with the known safety profile of teplizumab. Importantly, although the COVID-19 pandemic interfered with the overall conduct of the study, we did not find a difference in the rates of COVID-19 infection or other infections in the ITT population, further reinforcing the immuno-modulating action of teplizumab rather than an immuno-suppressive effect, which is a potential concern with other immune therapies that have been recently tested in patients with type 1 diabetes [20–22].

In conclusion, this analysis of participants who completed teplizumab treatment according to the study protocol showed that teplizumab can be administered to children and adolescents as an immuno-modulatory therapy. Those who received the protocol-specified teplizumab dosing regimen had greater beta cell function preservation and improved glucose management, as seen by the greater TIR and lower exogenous insulin use compared with placebo.

## Supplementary Information

Below is the link to the electronic supplementary material.

Supplementary file1 (PDF 384 KB)

## Data Availability

Qualified researchers may request access to participant-level data and related documents. Participant-level data will be anonymised, and study documents will be redacted to protect the privacy of trial participants. Further details on Sanofi’s data sharing criteria, eligible studies, and process for requesting access can be found at https://www.vivli.org/.

## Abbreviations

AE: Adverse event
AUC: Area under the concentration–time curve
CGM: Continuous glucose monitoring
eDiary: Electronic diary
GAD65: Glutamic acid decarboxylase 65
HLA: Human leukocyte antigen
ICA: Islet cell cytoplasmic autoantibody
ITT: Intent to treat
LS: Least squares
MMRM: Mixed-effect model for repeated measures
PP: Per protocol
TIR: Time in range

## Authors’ relationships and activities

KCH has acted as a consultant for Provention Bio and has received a contract from Intrexon. KCH is a co‑inventor on a patent for the use of teplizumab for delay of diagnosis of stage 3 type 1 diabetes. CMD has lectured for or been involved as an advisor to Novo Nordisk, Sanofi-Genzyme, Janssen, Servier, Lilly, AstraZeneca, Provention Bio, UCB, MSD, Vielo Bio, Avotres, Worg and Novartis. CMD holds a patent jointly with Midatech plc and Provention Bio. SEG reports consultant fees from Abata Therapeutics, Eli Lilly, Genentech, Gentibio Inc, SAB Biotherapeutics, Sana Biotechnology and Sanofi, and a data and safety monitoring fee from Diamyd Medical. ZS reports speakers’ honoraria from Medtronic, Abbott and A-Import. KS has acted as a consultant for Provention Bio. AS reports speakers’ honoraria and being involved as an advisor to Sanofi. LAK is an employee of Sanofi and may hold Sanofi stock/shares. EN is an employee of Sanofi and may hold Sanofi stock/shares. MRR was an employee of Provention Bio, a Sanofi company. WT was an employee of Provention Bio, a Sanofi company. RH was an employee of Sanofi at the time of the study and may hold Sanofi stock/shares. ELR was an employee of Provention Bio, a Sanofi company, and may hold Sanofi stock/shares. LC declares that there are no relationships or activities that might bias, or be perceived to bias, their work.
