# Supplementary material for: Preserving beta cell function in children and adolescents with newly diagnosed stage 3 type 1 diabetes: per-protocol population analysis from the PROTECT randomised trial
Source: Diabetologia. 2026 Sep 10;69(12):3501–11. doi: 10.1007/s00125-026-06849-5 (PMC13714163; doi:10.1007/s00125-026-06849-5)
Supplement: Supplementary file 1 [file 125_2026_6849_MOESM1_ESM.pdf]

Electronic supplementary material

ESM Table 1. Summary of participants in the ITT population excluded from the PP population analysis

**n (%)**	**Teplizumab (N=217)**	**Placebo (N=111)**
**PP population**	**180 (83%)**	**95 (86%)**
**Proportion of ITT population excluded**	**37 (17%)**	**16 (14%)**
**Reasons for exclusion**		
Treatment adherence <80%	32 (15)	15 (14)
AE	15	3
CRS grade 1	2	0
CRS grade 3	2	0
Elevated LFT	9	1
COVID-19	1	1
Cellulitis left arm, IV line-related bacteraemia	0	1
Delayed hypersensitivity reaction	1	0
Phlebitis right midline	1	0
Withdrawal of consent/patient decision	12	11
Dosing error[a]	2	0
Unable to come for infusion	1	1[b]
Live vaccine by paediatrician	1	0
Pregnancy	1	0
Took prohibited medication	3 (1)	1 (1)
Concomitant solumedrol treatment	1	0
Dexamethasone treatment	0	1
Extensive steroid treatment	1[c]	0
Metformin treatment	1	0
Received incorrect treatment allocation	2 (1)	0
Incorrect assigned vial administered[a]	2	0
Pregnancy	1 (<1)	0

AE, adverse event; C1, course 1; C2, course 2; CRS, cytokine release syndrome; ITT, intent-to-treat; IV, intravenous; LFT, liver function test; PP, per protocol.

[a]Two participants received 1 day of placebo in place of teplizumab; both participants received 23 of 24 days of teplizumab treatment.

[b]Reason listed as "other: consented on Modified Dosing Schedule; however, unable to perform course 2 due to local COVID rates".

[c]Participant was treated with extensive steroids during both C1 (methylprednisolone IV on dosing days 2–6) and C2 (prednisone oral on dosing days 1–12) to treat CRS.

ESM Figure 1. CONSORT diagram of patients in PROTECT

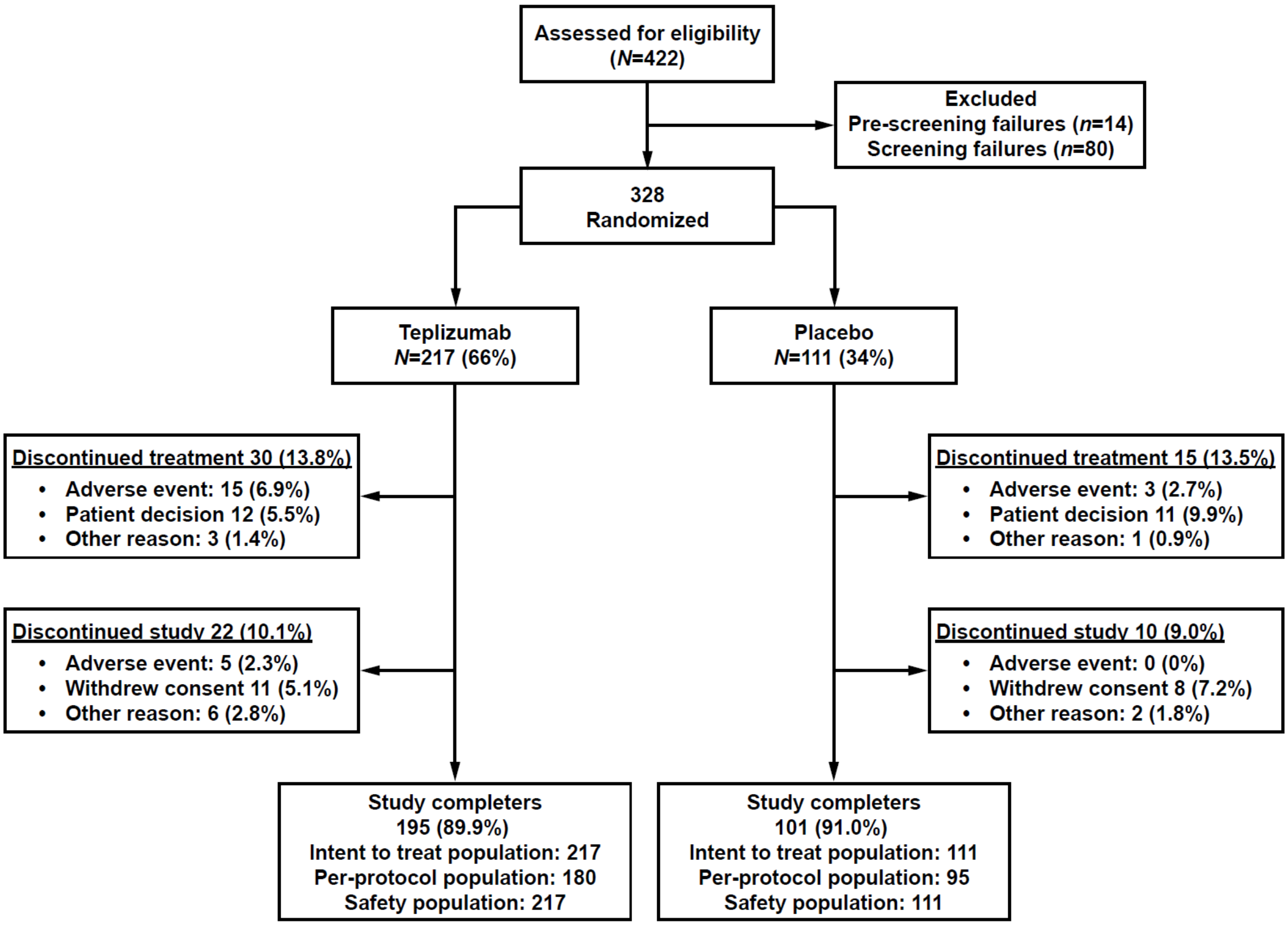

Adapted from Ramos EL, Dayan CM, Chatenoud L, et al. (2023) Teplizumab and β-cell function in newly diagnosed type 1 diabetes. N Engl J Med 389(23): 2151–2161. 10.1056/NEJMoa2308743

ESM Figure 2. Lymphocyte count (10^{-9}/l) over time during the dosing period and through treatment course 1 (a) and 2 (b) (PP Population)

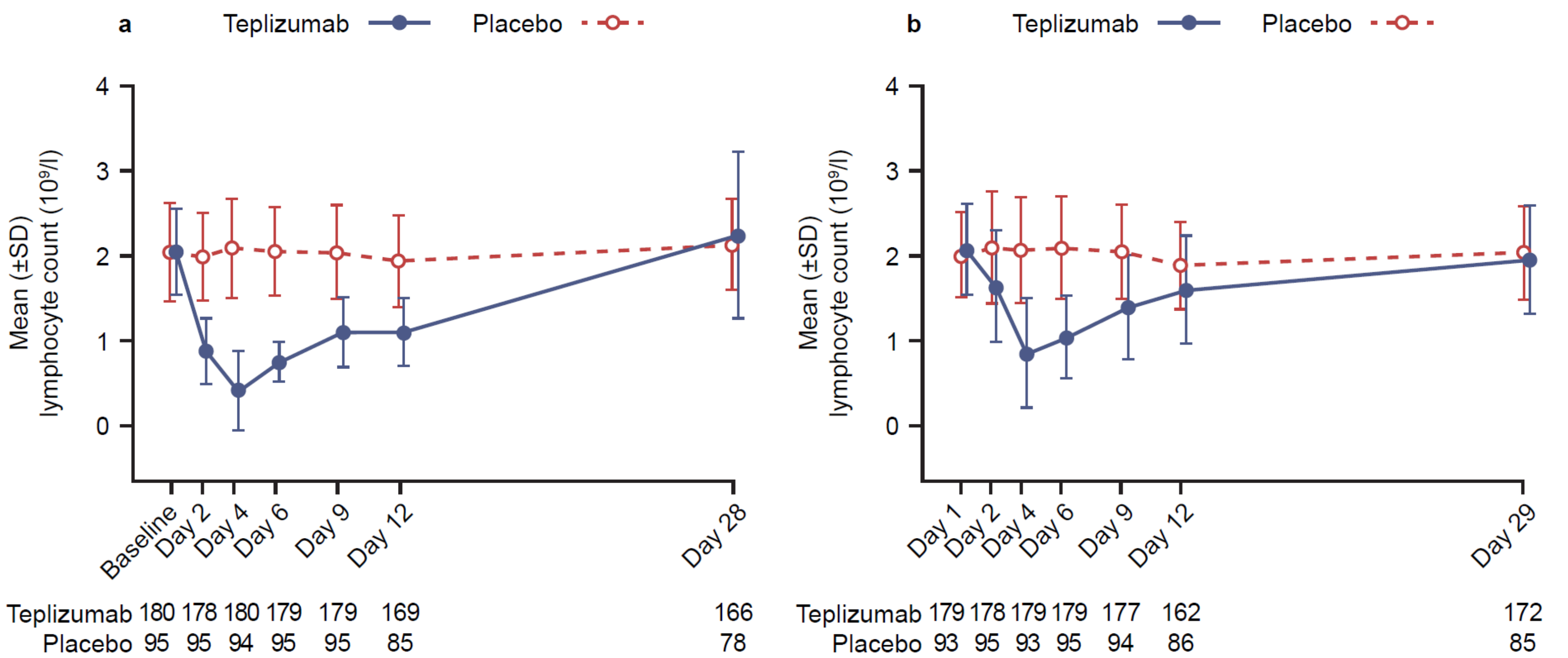

PP, per protocol; SD, standard deviation.

Within each treatment course, the lab sample was taken prior to dosing for baseline/day 1, day 2, day 4, day 9, and day 12. For course 2, data were collected on days 182, 183, 185, 187, 190, 193, and 210 for patients on the normal dosing schedule and on days 364, 365, 367, 369, 372, 375, and 392 for patients on the modified dosing schedule.
